# Abdominal pedunculated accessory liver lobe in a dog

**DOI:** 10.1111/jsap.70118

**Published:** 2026-03-15

**Authors:** G. Bison, T. Banzato, A. Zotti

**Affiliations:** ^1^ Department of Animal Medicine, Production and Health University of Padua Padua Italy

An 8‐year‐old spayed female mixed‐breed dog was presented with dysorexia, vomiting and diarrhoea lasting 10 days. Physical examination, haematology and serum biochemistry were unremarkable except for a mild hypoglycaemia and alkalosis. An abdominal ultrasound revealed no specific gastrointestinal pathological features but an unusual perisplenic parenchymal structure. The mass was clearly separable from the splenic tail, showing a coarser texture than that of the spleen and a mixed echogenicity due to the presence of both hyper‐ and hypo‐echoic areas. A vascular pedicle connecting the left lateral liver lobe to the mass was hypothesised (Fig 1A) and confirmed by colour Doppler ultrasound (Fig 1B). A tentative diagnosis of a degenerated pedunculated accessory liver lobe was made; the CT confirmed both the perisplenic soft tissue structure with attenuation and contrast enhancement similar to that of liver except for a focal hypodense area and the vascular pedicle (Fig 1C). Fine‐needle aspirate of the mass was obtained and cytology was consistent with hepatocellular hyperplasia with moderate‐to‐high degeneration. The gastroenteric symptoms resolution with supportive treatment coupled with the accessory liver lobe hepatocellular degeneration benign nature and the absence of relevant blood analysis abnormalities led to the patient discharge upon owner's decision. Accessory lobes are well differentiated from ectopic liver tissue in the human medical literature and pedunculated accessory lobes are defined as such if a pedicle is present. To our knowledge, this is the first description of a non‐complicated abdominal degenerated accessory liver lobe joined to the left lateral hepatic lobe with a vascular pedicle.

**FIG 1:**
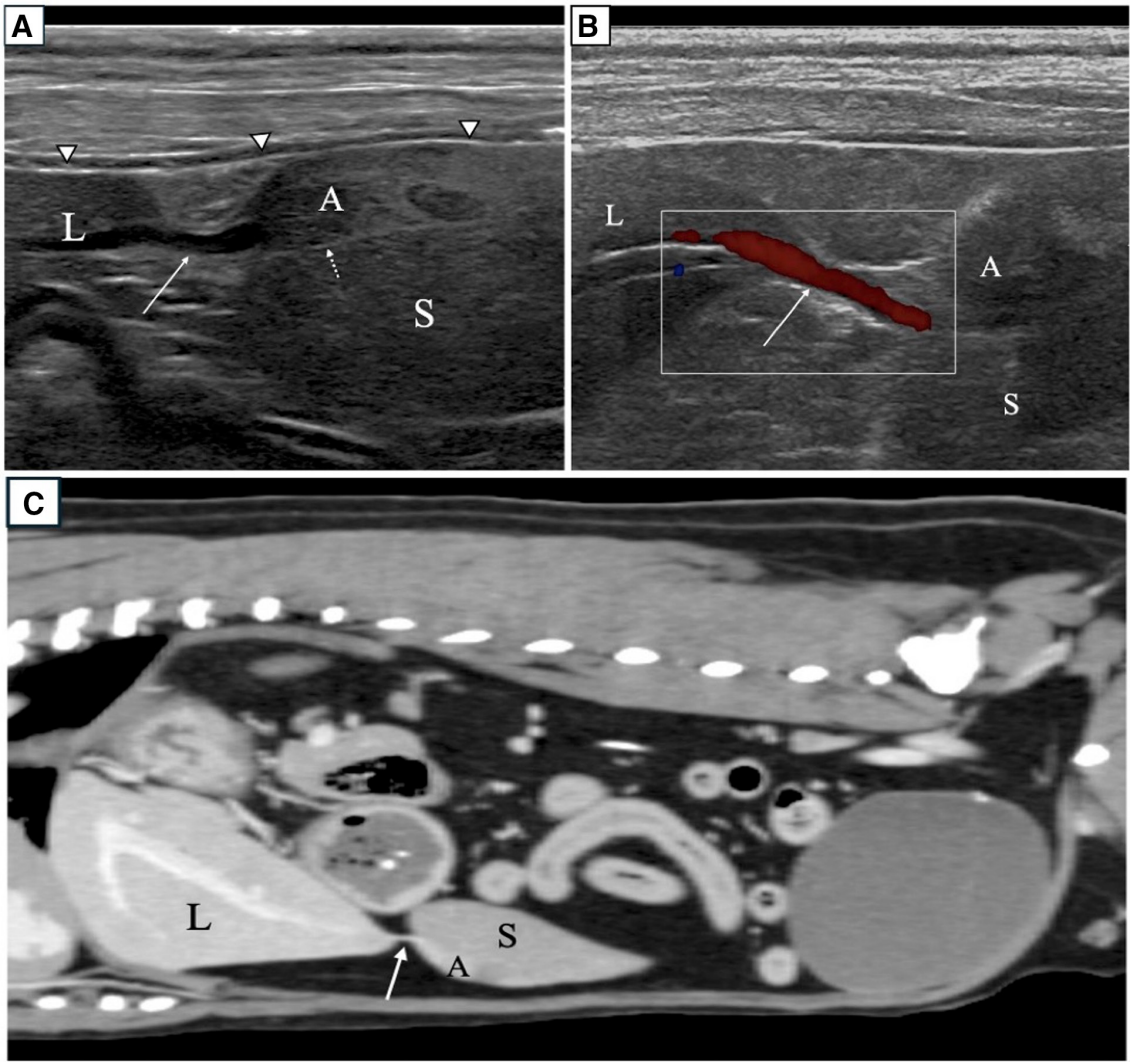
In all images, accessory liver lobe is labelled as “A”, the spleen as “S”, the liver as “L” and the pedicle as an “arrow”. (A) Dorsal ultrasonographic view of the accessory liver lobe appearing connected to the liver by a pedicle; the structure is located between the left abdominal wall (arrowheads) and the spleen, clearly separable from the splenic tail (dashed arrow). (B) Colour Doppler ultrasound image depicting the vessel within the pedicle. (C) Sagittal post‐contrast CT image showing the vascular pedicle between the liver and the accessory liver lobe.

## Author contributions

G.B. contributed to data collection (clinical investigation) and writing. T.B. and A.Z. contributed to data collection and writing. G.B. contributed to data collection, writing and revision of the manuscript.

